# Involvement of TGFβ-Induced Phosphorylation of the PTEN C-Terminus on TGFβ-Induced Acquisition of Malignant Phenotypes in Lung Cancer Cells

**DOI:** 10.1371/journal.pone.0081133

**Published:** 2013-11-22

**Authors:** Daisuke Aoyama, Naozumi Hashimoto, Koji Sakamoto, Takashi Kohnoh, Masaaki Kusunose, Motohiro Kimura, Ryo Ogata, Kazuyoshi Imaizumi, Tsutomu Kawabe, Yoshinori Hasegawa

**Affiliations:** 1 Department of Respiratory Medicine, Nagoya University Graduate School of Medicine, Nagoya, Japan; 2 Department of Respiratory Medicine and Allergy, Fujita Health University, Toyoake, Japan; 3 Department of Medical Technology, Nagoya University Graduate School of Health Science, Nagoya, Japan; University of Illinois at Chicago, United States of America

## Abstract

Transforming growth factor β (TGFβ) derived from the tumor microenvironment induces malignant phenotypes such as epithelial-mesenchymal transition (EMT) and aberrant cell motility in lung cancers. TGFβ-induced translocation of β-catenin from E-cadherin complexes into the cytoplasm is involved in the transcription of EMT target genes. PTEN (phosphatase and tensin homologue deleted from chromosome 10) is known to exert phosphatase activity by binding to E-cadherin complexes via β-catenin, and recent studies suggest that phosphorylation of the PTEN C-terminus tail might cause loss of this PTEN phosphatase activity. However, whether TGFβ can modulate both β-catenin translocation and PTEN phosphatase activity via phosphorylation of the PTEN C-terminus remains elusive. Furthermore, the role of phosphorylation of the PTEN C-terminus in TGFβ-induced malignant phenotypes has not been evaluated. To investigate whether modulation of phosphorylation of the PTEN C-terminus can regulate malignant phenotypes, here we established lung cancer cells expressing PTEN protein with mutation of phosphorylation sites in the PTEN C-terminus (PTEN4A). We found that TGFβ stimulation yielded a two-fold increase in the phosphorylated -PTEN/PTEN ratio. Expression of PTEN4A repressed TGFβ-induced EMT and cell motility even after snail expression. Our data showed that PTEN4A might repress EMT through complete blockade of β-catenin translocation into the cytoplasm, besides the inhibitory effect of PTEN4A on TGFβ-induced activation of smad-independent signaling pathways. In a xenograft model, the tumor growth ratio was repressed in cells expressing PTEN4A. Taken together, these data suggest that phosphorylation sites in the PTEN C-terminus might be a therapeutic target for TGFβ-induced malignant phenotypes in lung cancer cells.

## Introduction

Mounting evidence suggests the importance of the tumor microenvironment in which lung cancer cells interact with carcinoma-associated fibroblasts (CAFs) and the extracellular matrix (ECM) and consequently acquire various malignant phenotypes including epithelial-mesenchymal transition (EMT) and aberrant cell motility [1,2]. Transforming growth factor β (TGFβ), one of the most critical tissue-stiffening factors derived from the tumor microenvironment, causes the acquisition of malignant phenotypes, accompanied by the altered expression of EMT-related genes such as snail [3]. A recent study suggests that TGFβ-induced transcription of EMT target genes such as fibronectin and vimentin is accelerated by translocation of β-catenin from E-cadherin complexes at the cell membrane into the cytoplasm [4]. TGFβ stimulation also causes aberrant cell motility though smad-independent pathways, such as those involving focal adhesion kinase (FAK) and phosphatidylinositol-3-kinase (PI3K) [5,6]. Although many smad-independent pathways in the tumor microenvironment are negatively regulated by the concerted lipid and protein phosphatase activities of PTEN (phosphatase and tensin homologue deleted from chromosome 10) [7], lung cancers, in which mutation of the PTEN gene is rarely observed [8,9], often show hyperactivation of these pathways [9-11]. Although PTEN exerts its phosphatase activity by binding to E-cadherin complexes via β-catenin [12], recent studies have suggested that phosphorylation of the PTEN C-terminal tail might be closely associated with the loss of PTEN activity [13]. Rahdar et al. suggested that substitution with four alanine (Ala) residues, resulting in elimination of the corresponding serine/threonine phosphorylation sites (S380A, T382A, T383A, and S385A), enhanced membrane association of PTEN with an open conformation [14]. Some signaling pathways can modulate PTEN expression, resulting in decreased PTEN phosphatase activity [15,16]; however, whether TGFβ can modulate both β-catenin translocation and PTEN phosphatase activity via phosphorylation of the PTEN C-terminus remains elusive. Furthermore, the exact role of phosphorylation of the PTEN C-terminus in TGFβ-induced EMT and aberrant cell motility has not fully been evaluated. In the present study, we investigated whether TGFβ can modulate phosphorylation of the PTEN C-terminus in lung cancer cells and whether four-Ala substitution on the PTEN C- terminus (PTEN4A) could inhibit TGFβ-induced EMT and the related aberrant cell motility. Furthermore, we examined the underlying mechanism-that is, whether PTEN4A can modulate cadherin junctional complexes and signaling pathways. We also evaluated the effect of the compensatory induction of PTEN4A on tumor growth *in vivo*. 

## Materials and Methods

### Ethical Statement

All animal studies have been reviewed and approved by the University Committee on Use and Care of Animals at Nagoya University Graduate School of Medicine.

They were also conducted in accordance with institutional guidelines, and all efforts were made to minimize suffering. All mice were housed individually in a sterile barrier facility with fade-in/fade-out 12 hours light: 12 hours darkness. When mice were sacrificed after the experiments, mice were euthanized with anesthetic overdose, followed by immediate cervical dislocation, to minimize suffering. 

### Materials

Monoclonal mouse anti-PTEN antibody (clone 6H2.1) was from Cascade Bioscience (Winchester. United Kingdom). Purified rabbit anti-phospho-PTEN (Ser380/Thr382/Thr383) antibody, rabbit anti-pan Akt antibody, rabbit anti-phospho-Akt (Thr308) antibody, rabbit anti-phospho-Akt (Ser473) antibody, rabbit anti-FAK antibody, rabbit anti-phospho-FAK (Tyr397) antibody, mouse anti-smad2 antibody, and rabbit anti-phospho-smad2 (Ser465/467) antibody were from Cell Signaling Technology (Boston, MA). Purified anti-fibronectin antibody was from Santa Cruz Biotechnology, Inc (Santa Cruz, CA). Purified mouse anti-E-cadherin antibody and anti-β-catenin antibody were from BD Biosciences (San Diego, CA). Streptavidin (SAv)-Alexa 594 (SAv-594)-conjugated anti mouse antibody was from Invitrogen Life Technologies (Carlsbad, CA). Monoclonal mouse anti-vimentin antibody was from Millipore (Cambridge, United Kingdom). Affinity-isolated rabbit anti-actin antibody and SB 431542, a potent inhibitor of TGFβ type I receptor kinases, were from Sigma-Aldrich (St. Louis, MO). Can Get Signal was from Toyobo Co. (Tokyo, Japan). Doxycycline was from Clontech (Mountain View, CA). PhosSTOP and WST-1 were from Roche Applied Science (Mannheim, Germany). Hoechst33342 was from Dojindo (Kumamoto, Japan). 1,2,4,5-Benzenetetramine tetrahydrochloride (FAK inhibitor 14) was from Tocris Bioscience (Bristol, United Kingdom). 

### Plasmids and gene transfection

Human PTEN (NM_000314) cDNA was subcloned into the pEGFP-C1 vector (Clontech; Mountain View, CA). GFP or a fusion-gene of GFP-PTEN was placed into the pTRE-Tight vector (Clontech; Mountain View, CA), respectively. The QuikChange Site-Directed Mutagenesis Kit (Stratagene; La Jolla, CA) was utilized for the establishment of four-Ala substitution (S380A, T382A, T383A, and S385A) on the PTEN C-terminal tail (PTEN4A). Lastly, GFP in the pTRE-Tight vector (GFP), GFP-PTEN in pTRE-Tight vector (GFPPTENWt), and GFP-PTEN4A in the pTRE-Tight Vector (GFPPTEN4A) were established in this study. After the establishment of H358 cells, the human lung cancer cell line, carrying pTet-On Advanced (H358ON), GFP, GFP-PTENWt, or GFP-PTEN4A was co-transfected with the Linear Hygromycin Marker (Clontech; Mountain View, CA), by using Nucleofector™ II (Amaxa Biosystems, Gaithersburg, MD). After selection with hygromycin, single clones were isolated. Human PTEN was also placed into pcDNA4 (Invitrogen Life Technologies; Carlsbad, CA). H1299 cells, the other human lung cancer cell line, were electroporated with pcDNA4 only (4HC), pcDNA4 with PTENWt (PTENWt) or pcDNA4 with PTEN4A (PTEN4A) by using Nucleofector™ II. After selection with zeocin, single clones were isolated.

### Cells

The human lung cell lines, H358 and H1299, were maintained in RPMI supplemented with 2mmol/L L-glutamine, 100U/mL penicillin, 100μg/mL streptomycin, 0.25μg/mL fungizone, and 10%FCS [17]. To evaluate the effect of TGFβ on these cells, the cells were treated with TGFβ at 2 ng/ml and analyzed for mRNA levels and protein levels at the indicated time points and as described below. To evaluate the effect of PTEN transduction on TGFβ stimulation, H358ON cells expressing Dox-dependent GFP, GFP-PTENWt, or GFP-PTEN4A were incubated with Dox at 1 μg/ml for 24 hours before TGFβ treatment as described above. To examine the direct effects of TGFβ stimulation on modulation of the p-PTEN/PTEN ratio, cells were incubated with vehicle or SB 431542 for 1 hour before TGFβ treatment. To examine the role of FAK phosphorylation on TGFβ-induced EMT, the cells were incubated with vehicle or FAK inhibitor 14 for 24 hours before TGFβ treatment. 

### Cell migration assay

A 8-μm pore-size Boyden chamber was used for the in vitro migration assay [18]. After being treated with Dox for 24 hours, the cells (3x10^4^ cells) in RPMI medium containing 0.5% serum and TGFβ at 2 ng/ml were plated in the upper chamber and 15% fetal bovine serum (FCS) in RPMI was added to the lower chamber as a chemoattractant. 

### PCR analysis for expression of targeted genes

Real-time PCR was performed by using a TaqMan ABI 7300 Sequence Detection System (PE Applied Biosystems, Foster City, CA). Snail (NM_005985), twist (twist1: NM_000474), and glyceraldehydes-3-phophate dehydrogenase (GAPDH) mRNAs were detected, by using a mixture of oligonucleotide primers and probes from Nippon EGT, Inc (Toyama, Japan). mRNA levels were normalized to GAPDH mRNA signal [19]. 

### Western blot analysis

For whole-cell extracts, cells were harvested in ice-cold lysis buffer and cleared by centrifugation [20,21]. The samples were then subjected to SDSPAGE and analyzed by immunoblotting. To detect phosphorylation levels of the targeted proteins, Phosphostop was added to the lysis buffer and Can Get Signal was also added to the dilution solution for primary antibody and secondary antibody. β-actin was evaluated as a loading control.

### WST-1 assay

The cell proliferation reagent WST-1 was utilized for the quantitative determination of cell proliferation [18]. The absorbance of the samples was measured at 450 nm by using a spectrofluorophotometer (Wallac 1420 ARVO-SX; PerkinElmer, Inc., Waltham, MA). Unless otherwise noted, medium alone was measured as a background control.

### Immunofluorescence and confocal laser scanning microscopy

Immunochtochemistry was performed as previously reported [22]. To evaluate the effect of PTEN4A transduction on TGFβ-induced translocation of β-catenin, H358ON cells expressing Dox-dependent GFP, GFP-PTENWt, or GFP-PTEN4A were incubated with anti-β-catenin antibody followed by SAv-594 conjugated anti mouse antibody. Nuclear staining was performed by Hoechst 33342. To determine the levels of β-catenin distribution, confocal laser scanning microscopy (LSM 5 PASCAL; Carl Zeiss Co.,Ltd, JENA, Germany) was utilized. The fluorescence intensities of β-catenin and nucleus were evaluated by using imaging software (LSM Software ZEN 2008; Carl Zeiss Co.,Ltd, JENA, Germany). To perform the visual observation of the fluorescence, fluorescent intensities over a random cross section of the cells were plotted [23,24]. To determine the levels of PTEN subcellular distribution, confocal laser scanning microscopy was also utilized. The intensity levels of GFP fluorescence in both the cytoplasm and the nucleus were also quantified, by using the imaging software. A minimum of 5 randomly selected high-power fields were examined per sample to measure fluorescence intensity in the nucleus and the cytoplasm [25]. 

### Mouse xenograft model

3x10^6^ H358ON cells expressing Dox-dependent GFP, GFPPTENWt, or GFPPTEN4A, were inoculated subcutaneously (s.c.) into the flank of 6-week-old female nude mice and then maintained on water with Dox at final concentration 2mg/ml and autoclaved feed ad libitum. Growth was followed over time by taking caliper measurements at the indicated times as previously described [26,27]. Each experiment used 5 nude mice for GFPPTENWT, 7 nude mice for GFP, and 7 nude mice for GFPPTEN4A. Three independent experiments were performed. 

### Statistical analysis

The results were analyzed by using the Mann-Whitney test for comparison between any two groups, and by non-parametric equivalents of analysis of variance (ANOVA) for multiple comparisons. A value of p<0.05was considered to indicate statistical significance.

## Results

### TGFβ modulates phosphorylation levels of the PTEN C-terminus in PTEN expression in H358 cells, followed by EMT and aberrant cell motility

**Figure 1:**
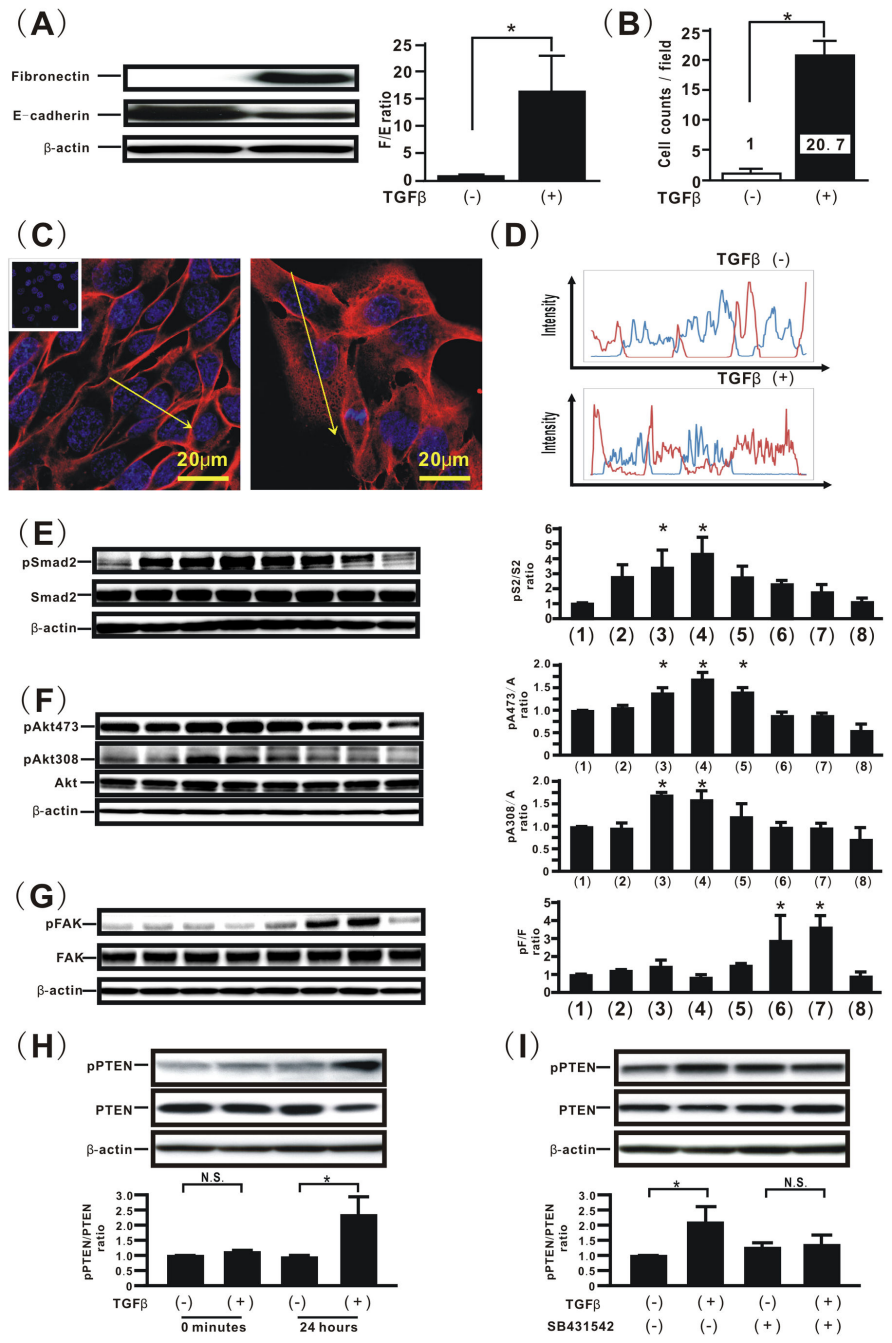
TGFβ modulates the phosphorylation levels of the PTEN C-terminus in PTEN expression in H358 cells, followed by EMT and aberrant cell motility. (**A**) H358 cells treated with vehicle or TGFβ were harvested for the analysis of fibronectin and E-cadherin. The relative expression of fibronectin to E-cadherin (F/E ratio) is shown in comparison to that in cells treated with vehicle. Data shown represent the means ± SE. The experiment was repeated three times with similar results. *: p<0.05 (**B**) A migration assay was performed for the H358 cell line treated with vehicle or TGFβ. Data shown represent the means ± SD. The experiment was repeated three times with similar results. *: p<0.05 The fluorescence intensities of β-catenin (red) in the treated cells were evaluated by using confocal laser scanning microscopy and imaging software. Nuclear staining was performed by Hoechst33342 (blue). The left image in (**C**) shows cells with no TGFβ stimulation. The right image in (**C**) shows cells stimulated with TGFβ. The cells incubated with isotype-matched control IgG is shown in the inset in (**C**). The upper panel in (**D**) plots the fluorescence intensity of β-catenin (red) and nucleus (blue) over a cross section of cells with no TGFβ stimulation. The lower panel in (**D**) plots the fluorescence intensity of β-catenin (red) and nucleus (blue) over a cross section of the cells stimulated with TGFβ. These figures are representative of at least three independent experiments. (**E**, **F**, and **G**) Cell extracts were harvested at the indicated periods after treatment with TGFβ for analysis of the levels of total and phosphorylated smad2 (**E**), Akt473 (**F**), Akt308 (**F**), and FAK (**G**). Results are shown for H358 naïve cells at 0 minutes (lane **1**), 5minutes (lane **2**), 20minutes (lane **3**), 1hour (lane **4**), 3hours (lane **5**), 6hours (lane **6**), 24hours (lane **7**), and 48hours (lane **8**) after treatment with TGFβ (left in E, F, and G). The ratio of phosphorylated protein to total protein is presented as the intensity level relative to that of H358 naïve cells at 0 minutes (lane **1**) after treatment with TGFβ (right in E, F, and G). Data shown represent the means ± SE. The experiment was repeated three times with similar results. *: p<0.05 (**H**) Cells treated with vehicle or TGFβ for 0 minutes or 24hours were harvested for the analysis of phosphorylated PTEN (pPTEN) and total PTEN. The relative expression of pPTEN to total PTEN (pPTEN/PTEN ratio) is shown in comparison to that in the cells treated with vehicle for 0 minutes. A representative blot from three independent experiments is shown. Data shown represent the means ± SE. The experiment was repeated three times with similar results. *: p<0.05 N.S. indicates “not significant”. (**I**) H358 naïve cells were incubated with vehicle or SB 431542 at 10 μM for one hour before TGFβ treatment. pPTEN/PTEN ratio is shown in comparison to that in cells treated with vehicle. A representative blot from three independent experiments is shown. Data shown represent the means ± SE. The experiment was repeated three times with similar results. *: p<0.05 N.S. indicates “not significant”.

To evaluate TGFβ-induced EMT in lung cancer cells [28,29], western blotting analysis for fibronectin [4,30] and E-cadherin [4,29] was performed. Western blotting analysis demonstrated that TGFβ treatment induced an approximately 12-fold increase in fibronectin expression in H358 naïve cells as compared with vehicle, whereas E-cadherin expression in cells treated with TGFβdecreased by more than 20% as compared with those treated with vehicle; thus, TGFβ stimulation yielded more than an 15-fold higher fibronectin/ E-cadherin ratio in H358 naïve cells as compared with vehicle (Figure 1A). To evaluate the association between TGFβ-induced EMT and migration ability, a migration assay was performed. H358 naïve cells treated with TGFβat 2 ng/ml exhibited an approximately 20-fold greater ability to migrate toward a chemoattractant, as compared with those treated with vehicle (Figure 1B). Recent studies suggest that translocation of β-catenin into the cytoplasm directly induces *de novo* expression of mesenchymal genes in epithelial cells [4,31]. Therefore, localization of β-catenin was also evaluated in TGFβ-treated lung cancer cells by immunofluorescence. Immunofluorescence images obtained by confocal microscopy suggested that β-catenin was localized on the cell membrane in H358 naïve cells treated with no TGFβ (Figure 1C and 1D), whereas β-catenin translocation into the cytoplasm was observed in TGFβ-treated H358 naïve cells, accompanied by co-localization of β-catenin with Hoechst33342 (Figure 1C and 1D). To evaluate the TGFβ-induced signaling pathways, western blotting was performed. TGFβ induced an increase in smad2 phosphorylation beginning at 5 minutes and reaching a maximum at 1 hour, after which phosphorylated smad2 expression was sustained at a steady level for up to 6 hours (Figure 1E). To evaluate the effect of TGFβ stimulation on smad-independent pathways, activation of Akt and FAK was also analyzed by western blotting. TGFβ treatment induced increasing phosphorylation of Akt at Thr308 and Ser473 (Akt308 and Akt473) beginning at 20 minutes and reaching a maximal level at 1 to 3 hours (Figure 1F); by contrast, increasing phosphorylation of FAK at Tyr397 was observed at 6 hours and reached a maximal level by 12 to 24 hours (Figure 1G). Furthermore, we evaluated the effect of TGFβ on both PTEN expression levels and phosphorylation of PTEN (p-PTEN) on its C-terminus in lung cancer cells. The results showed that TGFβ treatment slightly but substantially increased phosphorylation of PTEN on its C-terminus and also decreased total PTEN levels in H358 naïve cells, thus yielding a two-fold increase in the p-PTEN/PTEN ratio 24 hours after TGFβ treatment (Figure 1H). To evaluate whether TGFβ can directly modulate the p-PTEN/PTEN ratio, H358 cells were treated with SB 431542, a potent inhibitor of TGFβ type I receptor kinases [32]. Treatment with SB 431542 successfully inhibited the TGFβ-induced increase in the p-PTEN/PTEN ratio (Figure 1I), a finding supported by data showing that treatment with 10 μM SB 431542 inhibited smad2 activation (data not shown). Thus, a TGFβ-induced increase in the p-PTEN/PTEN ratio might be involved in the TGFβ-induced acquisition of malignant phenotypes in lung cancer cells. 

### Mutation of phosphorylation sites in the PTEN C-terminus represses TGFβ-induced EMT and aberrance cell motility in H358 cells

**Figure 2:**
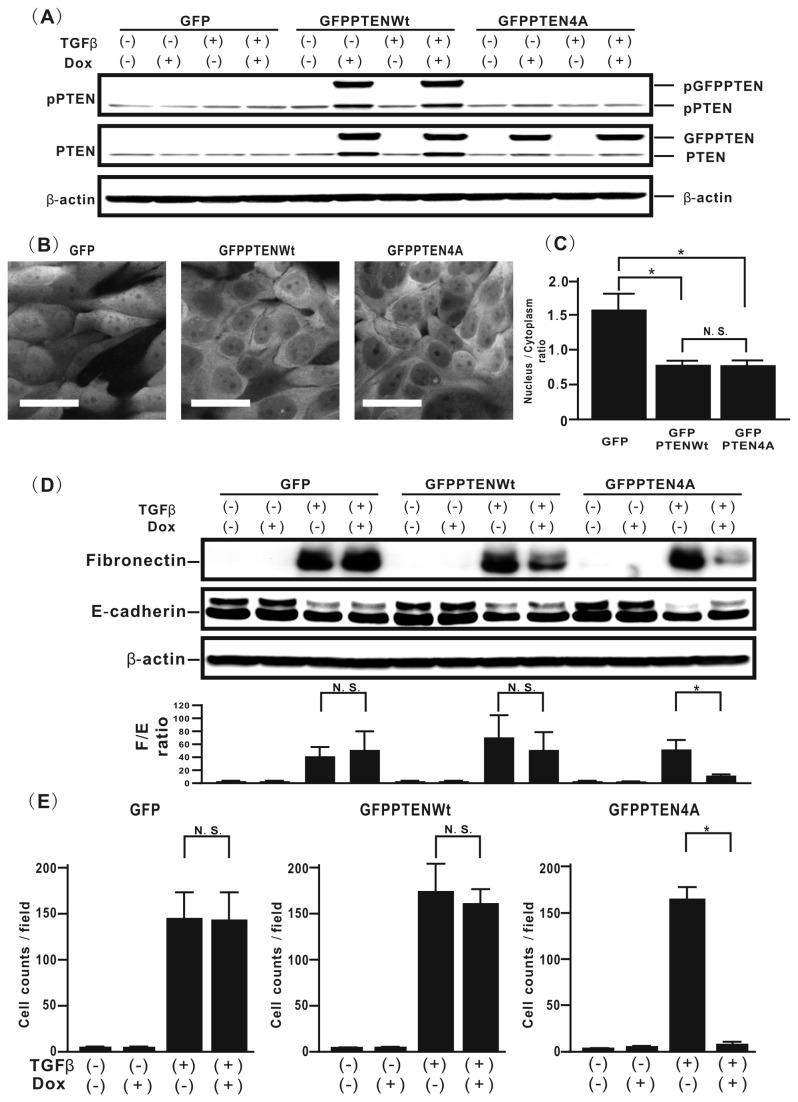
Mutation of phosphorylation sites in the PTEN C-terminus blocks TGFβ-induced EMT and aberrance cell motility in H358 cells. (**A**) H358ON cells expressing Dox-dependent GFP, GFP-PTENWt, or GFP-PTEN4A were incubated with vehicle or Dox for 24hours before TGFβ treatment. The cells were then treated with vehicle or TGFβ for a further 24hours in the absence or presence of Dox. The cells were harvested for the analysis of pPTEN (**top** panel), total PTEN (**middle** panel) and β-actin (**bottom** panel) by western blotting. A representative blot from three independent experiments is shown. (**B**) By using confocal laser scanning microscopy, the localization of GFP fluorescence in H358ON cells expressing Dox-treated GFP (**left panel**), GFP-PTENWt (**middle panel**) and GFP-PTEN4A (**right panel**) was evaluated. (**C**) The intensity levels of GFP fluorescence in both the cytoplasm and the nucleus were also quantified, by Imaging software. The fluorescence intensity was expressed as the nucleus/cytoplasm ratio for each sample. Data shown represent the means ± SEM from three independent experiments. *: p<0.05 N.S. indicates “not significant”. (**D**) H358ON cells expressing Dox-dependent GFP, GFP-PTENWt, or GFP-PTEN4A were treated with vehicle or TGFβ for 48hours in the absence or presence of Dox, and then harvested for the analysis of fibronectin, E-cadherin, and β-actin by western blotting. The F/E ratio is shown in comparison to that in cells treated with vehicle in the absence of Dox. A representative blot from three independent experiments is shown. Data shown represent the means ± SE. The experiment was repeated three times with similar results. *: p<0.05 N.S. indicates “not significant”. (**E**) A migration assay was performed for H358ON cells expressing Dox-dependent GFP, GFP-PTENWt, or GFP-PTEN4A in the absence or presence of Dox and/or TGFβ stimulation. Data shown represent the means ± SD. The experiment was repeated three times with similar results. *: p<0.05 N.S. indicates “not significant”.

To confirm the biological effects of TGFβ-induced phosphorylation of the PTEN C-terminus in lung cancer cells, we investigated whether mutation of phosphorylation sites in PTEN can affect both TGFβ-induced EMT and the migration ability of lung cancer cells by using a Dox-dependent gene expression system because several PTENWt reconstitution models have suggested that PTENWt transduction might induce a slow growth ratio in glioma cells [15]. First to verify that four-Ala substitution inhibits phosphorylation of the PTEN C-terminus in *de novo* PTEN protein induced by Dox, western blotting was performed on H358ON cells expressing Dox-dependent GFP, GFP-PTENWt, or GFP-PTEN4A in the absence or presence of Dox. Although *de novo* GFP-PTEN expression was observed in H358ON cells expressing Dox-dependent GFP-PTENWt or GFP-PTEN4A when Dox was added, phosphorylated GFP-PTEN was detected only in H358ON cells expressing Dox-dependent GFP-PTENWt (Figure 2A). Because recent studies suggest that unphosphorylated PTEN might be subject to ubiquitination, resulting in translocation into the nucleus [21], we evaluated the localization of GFP fluorescence in H358ON cells expressing Dox-dependent GFP, GFP-PTENWt and GFP-PTEN4A. GFP alone was diffusely expressed in both the cytoplasm and the nucleus (left in Figure 2B and 2C), whereas GFP-PTENWt and GFP-PTEN4A were mainly located in the cytoplasm with faint nuclear expression (middle and right in Figure 2B, respectively, and 2C). There was no difference in the nucleus/cytoplasm ratio of GFP fluorescence between GFP-PTENWt and GFP-PTEN4A (Figure 2C). To evaluate whether or not TGFβ-induced EMT can be modulated by *de novo* GFP-PTEN expression, western blotting analysis of fibronectin and E-cadherin was performed. There was no reduction in the fibronectin/E-cadherin ratio in cells expressing GFP alone and a partial reduction (31.8%) in those expressing GFP-PTENWt; however, *de novo* GFP-PTEN4A protein induced by Dox caused a significant decrease of about 75% in the fibronectin/E-cadherin ratio (Figure 2D). To evaluate whether GFP-PTEN4A can affect TGFβ-induced cell motility, a migration assay was performed. In H358ON cells, the TGFβ-induced increase in migration toward a chemoattractant was not inhibited by either GFP or GFP-PTENWt protein induced by Dox, whereas *de novo* GFP-PTEN4A protein repressed cell migration induced by TGFβ stimulation (Figure 2E). These results indicate that inhibiting phosphorylation of the PTEN C-terminus can repress TGFβ-induced EMT and block aberrant cell motility in lung cancer cells, beyond the effect of PTEN transduction itself observed in cells expressing PTENWt. 

### Mutation of phosphorylation sites in the PTEN C-terminus inhibits TGFβ-induced smad-independent pathways, but not the smad-dependent pathway in H358 cells

**Figure 3:**
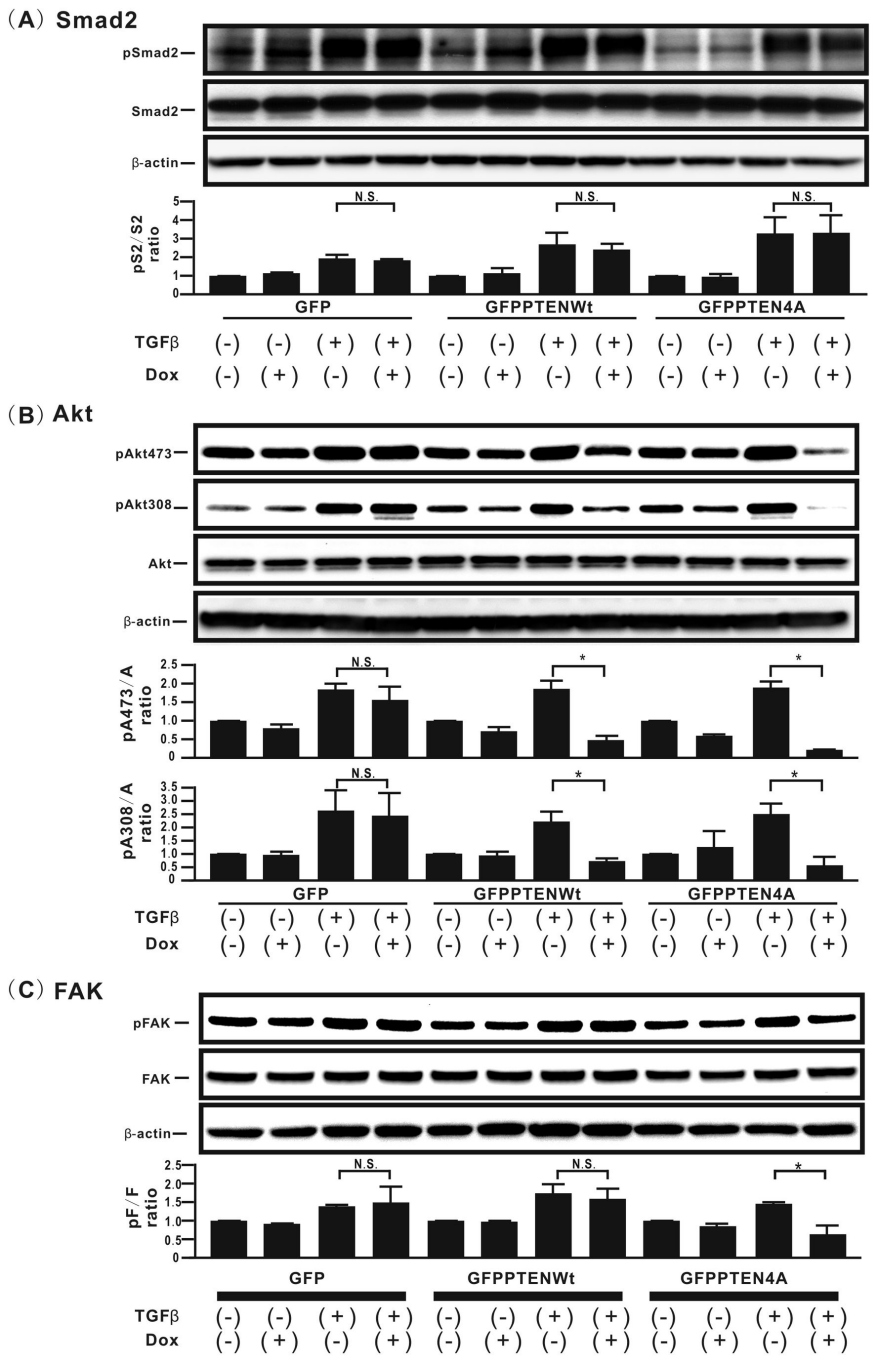
Mutation of phosphorylation sites in the PTEN C-terminus inhibits TGFβ-induced smad-independent pathways, but not the smad-dependent pathway in H358 cells. Cell extracts from H358ON cells expressing Dox-dependent GFP, GFP-PTENWt, or GFP-PTEN4A in the absence or presence of Dox were harvested for analysis of the levels of total and phosphorylated for smad2 (**A**), Akt473 (**B**), Akt308 (**B**), and FAK (**C**) at the indicated periods after treatment with vehicle or TGFβ (1hour for smad2, 1hour for Akt473, 1hour for Akt308, and 24hours for FAK, respectively). A representative blot from three independent experiments is shown (**top** in **A**, B and C). The ratio of phosphorylated protein to total protein is presented as the intensity level relative to that in H358ON cells expressing Dox-dependent GFP treated with vehicle in the absence of Dox (**bottom** in **A**, B and C). Data shown represent the means ± SE. The experiment was repeated three times with similar results. *: p<0.05 N.S. indicates “not significant”.

To elucidate the underlying molecular mechanisms, the effect of GFP-PTEN4A on TGFβ-induced signaling pathways was evaluated. Neither *de novo* GFP, GFP-PTENWt, nor GFP-PTEN4A expression induced by Dox repressed the increase in smad2 phosphorylation in TGFβ-treated H358ON cells (Figure 3A). The increase in Akt phosphorylation in TGFβ-treated H358ON cells expressing Dox-dependent GFP-PTENWt and GFP-PTEN4A returned to the basal level or lower when Dox was added, whereas that in TGFβ-treated H358ON cells expressing Dox-dependent GFP did not change when Dox was added (Figure 3B). It should be noted that GFP-PTEN4A steadily repressed phosphorylated Akt levels, as compared with GFP-PTENWt (GFP-PTEN4A, 88% decrease in Akt473 and 79% decrease in Akt308; GFP-PTENWt, 74% decrease in Akt473 and 68% decrease in Akt308) (Figure 3B). The level of phosphorylated FAK in TGFβ-treated H358ON cells expressing either GFP or GFP-PTENWt was not altered when Dox was added, whereas that in TGFβ-treated H358ON cells expressing GFP-PTEN4A successfully returned to the basal level when Dox was added (Figure 3C). 

### An FAK inhibitor targeting Tyr397 blocks TGFβ-induced aberrant cell motility, but not TGFβ-induced EMT in H358 cells

**Figure 4:**
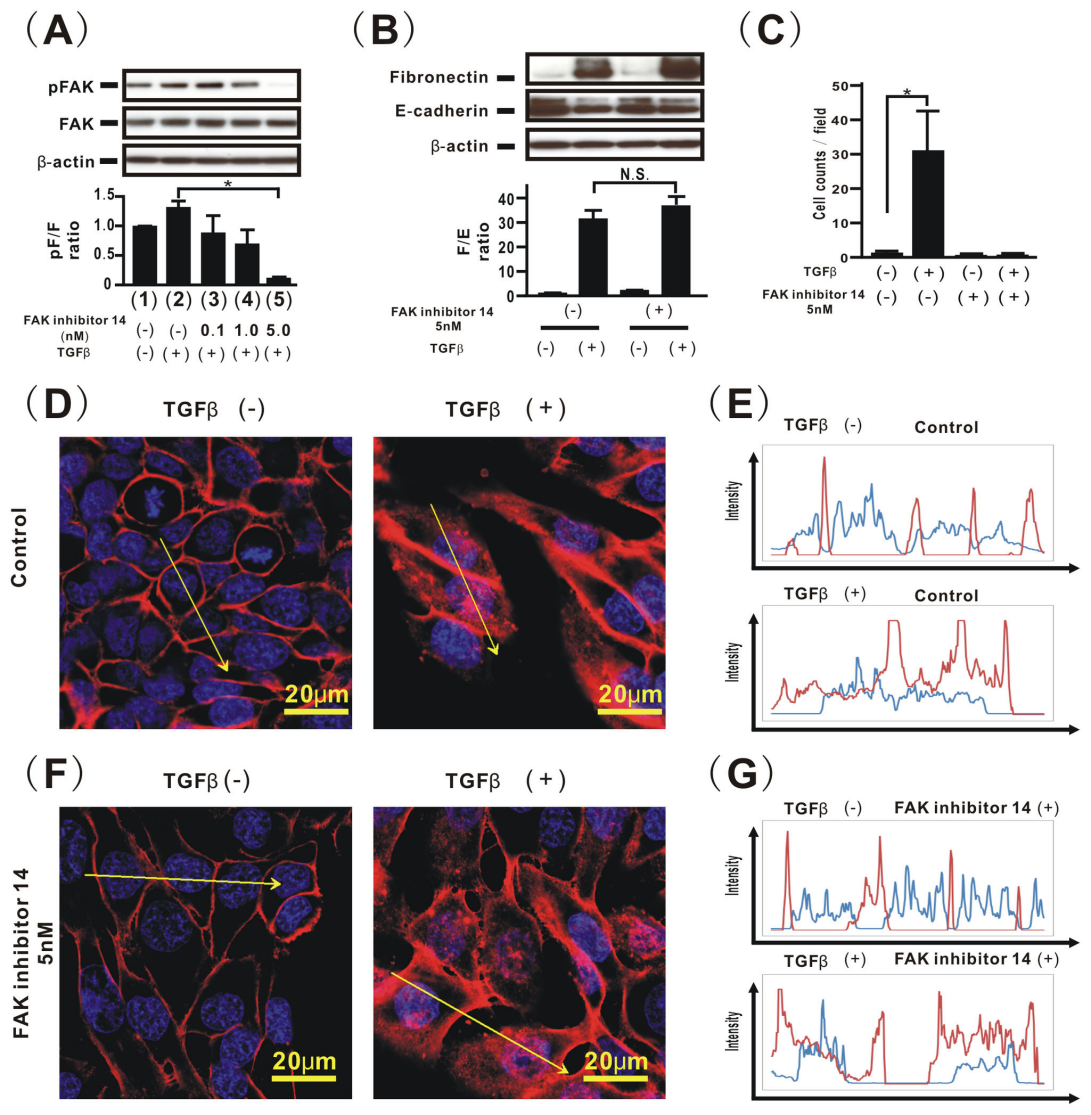
A FAK inhibitor targeting Tyr397 blocks TGFβ-induced aberrant cell motility, but not TGFβ-induced EMT in H358 cells. To examine the role of FAK phosphorylation at Tyr397 on TGFβ-induced EMT, Dox-treated H358ON cells expressing Dox-dependent GFP were incubated with vehicle or FAK inhibitor 14 for 24hours before TGFβ treatment. (**A**) Cell extracts were harvested 24 hours after treatment with TGFβ for analysis of the levels of total and phosphorylated FAK. Dox-treated H358ON cells expressing Dox-dependent GFP were treated with vehicle (lane **1**) or TGFβ (lane **2**, **3**, **4**, and **5**). The cells were also incubated with vehicle (lane **1** and **2**), or FAK inhibitor 14 at 0.1 nM (lane **3**), 1 nM (lane **4**), and 5 nM (lane **5**) (**top** in **A**). The ratio of phosphorylated protein to total protein is presented as the intensity level relative to that in Dox-treated H358ON cells expressing Dox-dependent GFP treated with vehicle (**bottom** in **A**). A representative blot from three independent experiments is shown. Data shown represent the means ± SE. The experiment was repeated three times with similar results. *: p<0.05 (**B**) Dox-treated H358ON cells expressing Dox-dependent GFP were treated with vehicle or TGFβ for 48hours in the absence or presence of FAK inhibitor 14 at 5nM, and then harvested for the analysis of fibronectin, E-cadherin, and β-actin by western blotting. The F/E ratio is shown in comparison to that in cells treated with vehicle (**bottom** in **B**). A representative blot from three independent experiments is shown. Data shown represent the means ± SE. The experiment was repeated three times with similar results. *: p<0.05 N.S. indicates “not significant”. (**C**) A migration assay was performed for Dox-treated H358ON cells expressing Dox-dependent GFP treated with vehicle or TGFβ for 48hours in the absence or presence of FAK inhibitor 14 at 5nM. Data shown represent the means ± SD. The experiment was repeated three times with similar results. *: p<0.05 To evaluate the effect of FAK inhibitor 14 on localization of β-catenin in Dox-treated H358ON cells expressing Dox-dependent GFP treated with vehicle or TGFβ, the intensities of fluorescence of β-catenin in the cells were evaluated. The cells were treated with vehicle (**D** and **E**) or FAK inhibitor at 5nM (**F** and **G**). The left image in (**D** and **F**) shows cells with no TGFβ stimulation. The right image in (**D** and **F**) shows cells stimulated with TGFβ. The cells incubated with isotype-matched control IgG is shown in the inset in (**D**). Each upper panel in (**E**) and (**G**) plots the fluorescence intensity of β-catenin (red) and nucleus (blue) over a cross section of cells with no TGFβ stimulation. Each lower panel in (**E**) and (**G**) plots the fluorescence intensity of β-catenin (red) and nucleus (blue) over a cross section of cells stimulated with TGFβ. These figures are representative of at least three independent experiments.

Because PTEN4A repressed the levels of phosphorylated FAK at Tyr397 induced by TGFβ, we determined whether inhibition of TGFβ-induced FAK activation can rescue EMT and block aberrant cell motilit. First, the cells were treated with FAK inhibitor 14, targeting the Tyr397 site of FAK [33], which successfully inhibited TGFβ-induced FAK phosphorylation at Tyr397 in a dose dependent manner (Figure 4A). Inhibition of TGFβ-induced FAK activity by FAK inhibitor 14 yielded repressed TGFβ-induced cell motility (Figure 4C), but it did not affect the acquisition of EMT phenotypes remained persistent (Figure 4B). Furthermore, we confirmed that TGFβ-induced β-catenin translocation into the cytoplasm and the nucleus was not blocked by treatment with FAK inhibitor 14 (Figure 4D-4G). 

Mutation of phosphorylation sites in the PTEN C-terminus inhibits TGFβ-induced EMT via blockade of β-catenin translocation, but not modulation of EMT-related genes in H358 cells.

**Figure 5:**
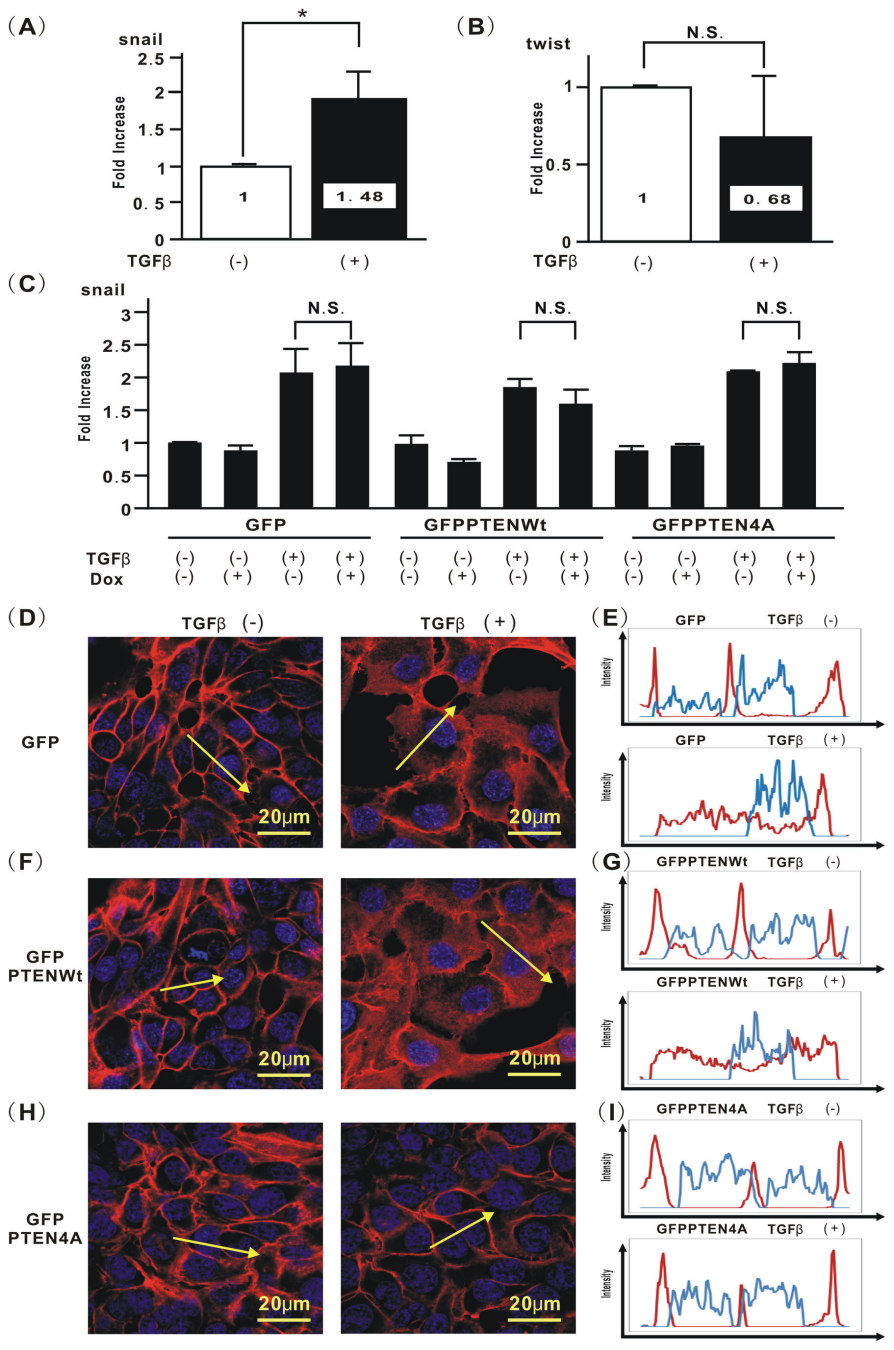
Mutation of phosphorylation sites in the PTEN C-terminus inhibits TGFβ-induced EMT via blockade of β-catenin translocation, but not by modulation of EMT-related genes in H358 cells. The expression levels of EMT-related genes were evaluated in H358 naïve cells treated with vehicle or TGFβ for 24hours. (**A**) snail mRNA and (**B**) twist mRNA were analyzed and normalized to GAPDH mRNA, by using real-time PCR. The relative expression of each targeted gene is shown in comparison to that in cells treated with vehicle. Data shown represent the means ± SEM from three independent experiments. *: p<0.05 N.S. indicates “not significant”. (**C**) H358ON cells expressing Dox-dependent GFP, GFP-PTENWt, or GFP-PTEN4A were incubated with vehicle or Dox for 24hours before TGFβ treatment. The cells were then treated with vehicle or TGFβ for a further 24hours in the absence or presence of Dox. Snail mRNA was analyzed and normalized to GAPDH mRNA, by using real-time PCR. The relative expression of the snail gene is shown in comparison to that in cells treated with vehicle in absence of Dox. Data shown represent the means ± SEM from three independent experiments. The fluorescence intensity of β-catenin in H358ON cells expressing Dox-dependent GFP (**D**), GFP-PTENWt (**F**), or GFP-PTEN4A (**H**) was evaluated. Each left image in (**D**), (**F**), and (**H**) shows cells with no TGFβ stimulation. Each right image in (**D**), (**F**), and (**H**) shows cells stimulated with TGFβ. Each upper panel in (**E**), (**G**), and (**I**) plotted the fluorescence intensity of β-catenin (red) and nucleus (blue) over a cross section of cells with no TGFβ stimulation. Each lower panel in (**E**), (**G**), and (**I**) plotted the fluorescence intensity of β-catenin (red) and nucleus (blue) over a cross section of cells stimulated with TGFβ. These figures are representative of at least three independent experiments.

To evaluate whether the inhibitory effect of mutation of phosphorylation sites in PTEN on TGFβ-induced EMT might be due to the altered expression of EMT-related genes, real-time PCR was performed. TGFβ stimulation induced an increase in snail expression in H358 cells (Figure 5A), but it did not appear to induce twist expression (Figure 5B). The increase in snail mRNA in TGFβ-treated H358ON cells expressing Dox-dependent GFP-PTEN4A did not change when Dox was added (Figure 5C), indicating that the inhibitory effect of PTEN4A on TGFβ-induced EMT might not be due to modulated expression of the snail gene. 

To determine whether or not TGFβ can modulate the β-catenin translocation via phosphorylation of the PTEN C-terminus, we evaluated the effect of compensatory induction of PTEN4A on β-catenin localization in TGFβ-treated lung cancer cells. Immunofluorescence images obtained by confocal microscopy suggested that β-catenin was localized on the cell membrane in H358ON cells expressing Dox-dependent GFP, GFP-PTENWt, or GFP-PTEN4A when no TGFβ was added (Figure 5D-5I). Whereas TGFβ-induced translocation of β-catenin into the cytoplasm and the nucleus in H358ON cells was not inhibited by either GFP or GFP-PTENWt protein induced by Dox, expression of only *de novo* GFP-PTEN4A protein completely retained localization of β-catenin on the cell membrane in H358ON cells after TGFβ stimulation (Figure 5D-5I). Taken together, these results show that PTEN4A, but not PTENWt, might rescue TGFβ-induced EMT via blockade of β-catenin translocation from the cell membrane into the cytoplasm.

### Mutation of phosphorylation sites in the PTEN C-terminus modulates TGFβ-induced cell proliferation in H358 cells

**Figure 6:**
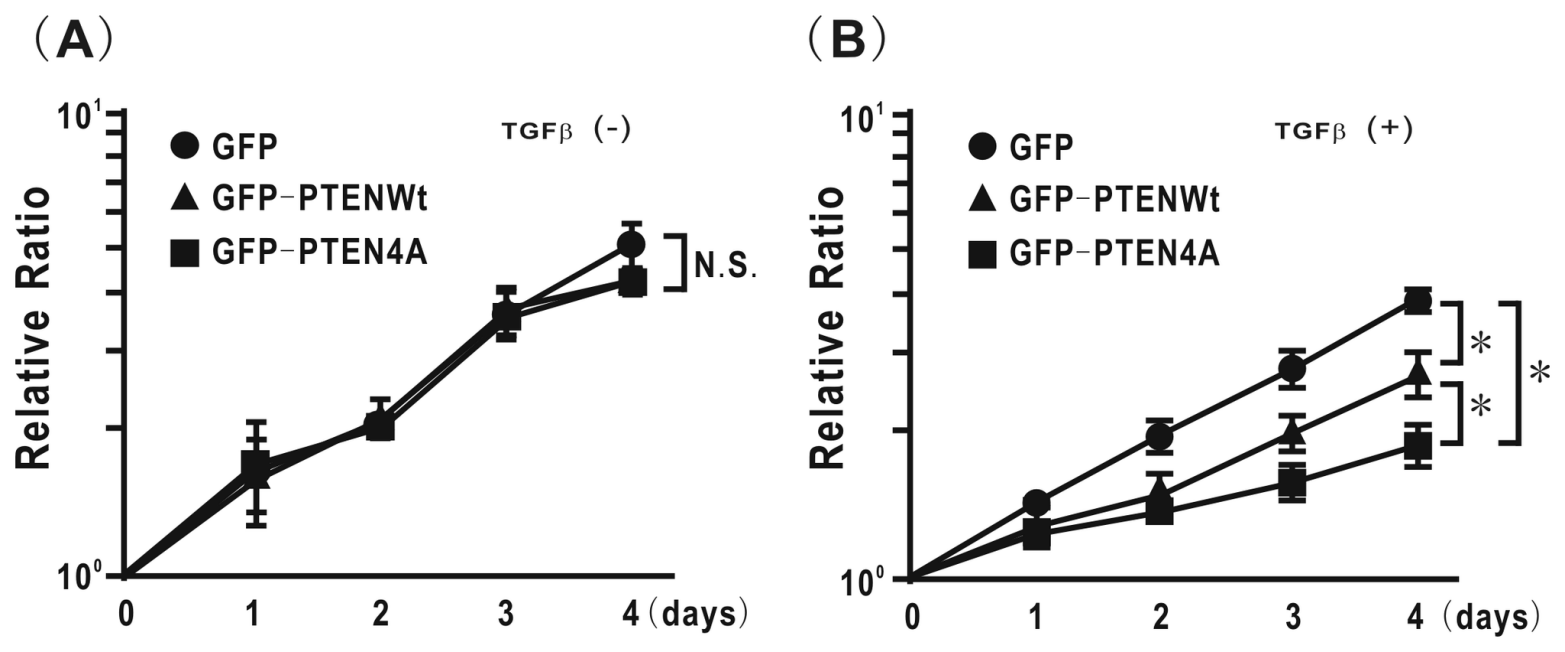
Mutation of phosphorylation sites in the PTEN C-terminus modulates TGFβ-induced cell proliferation in H358 cells. (**A**) Cell proliferation was measured by WST assay for H358ON cells expressing Dox-dependent GFP, GFP-PTENWt, or GFP-PTEN4A in the presence of Dox. The experiment was repeated three times with similar results. (**B**) Cell proliferation was also measured for H358ON cells expressing Dox-dependent GFP, GFP-PTENWt, or GFP-PTEN4A treated with TGFβ in the presence of Dox. The experiment was repeated three times with similar results. N.S. indicates “not significant”. *: p<0.05 .

To evaluate the effect of mutation of phosphorylation sites in PTEN on cell proliferation, a WST-1 assay was performed. Neither *de novo* GFP, GFP-PTENWt, nor GFP-PTEN4A expression induced by Dox affected cell proliferation ability in untreated cells with TGFβ (Figure 6A); by contrast, both GFP-PTENWt and GFP-PTEN4A induced a significant decrease in cell proliferation in cells treated with TGFβ as compared with GFP (Figure 6B). Because recent studies have reported that mutation of phosphorylation sites in PTEN favors nuclear accumulation of PTEN [21,34], we evaluated whether or not TGFβ can induce PTEN nuclear accumulation. However, TGFβstimulation of H358ON cells expressing Dox-dependent GFP, GFP-PTENWt and GFP-PTEN4A did not appear to modulate PTEN nuclear accumulation (data not shown).

### Mutation of phosphorylation sites in the PTEN C-terminus represses TGFβ-induced EMT and aberrance cell motility in H1299 lung cancer cells

**Figure 7:**
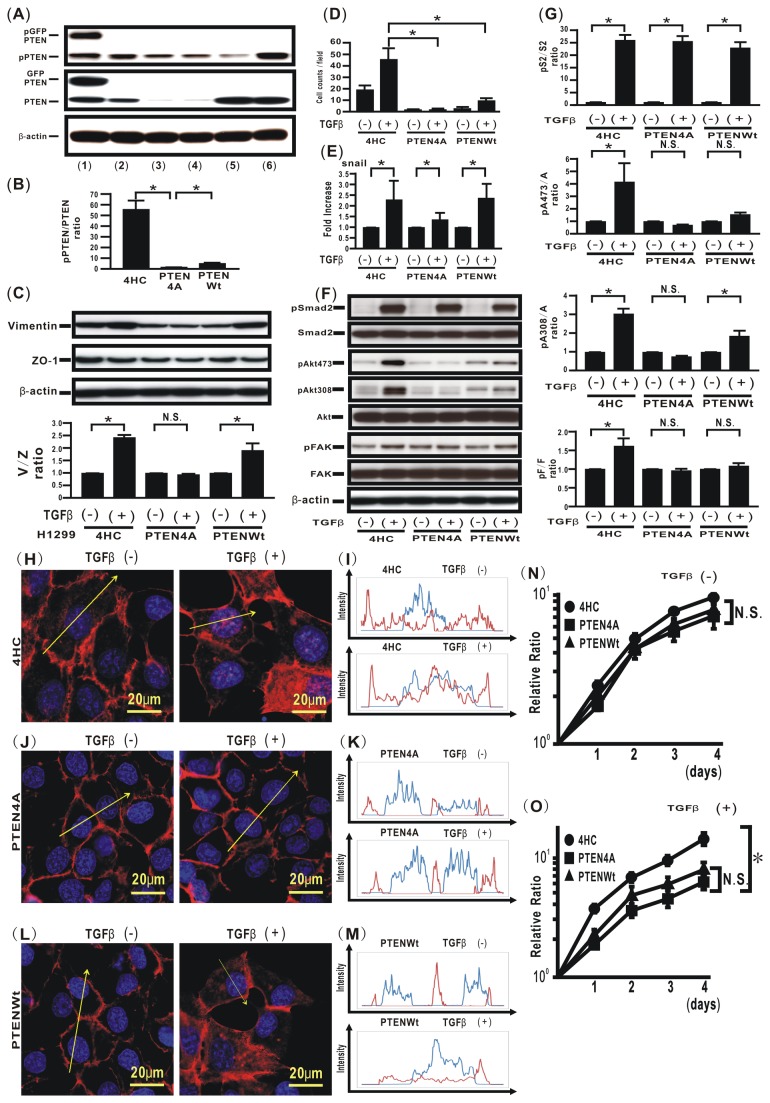
Compensatory induction of PTEN4A inhibits TGFβ-induced malignant phenotypes in H1299 cells. (**A**) Cell extracts were harvested for analysis of the levels of pPTEN (**top** panel), total PTEN (**middle** panel) and β-actin (**bottom** panel) by western blotting: H358ON cells expressing Dox-treated GFP-PTENWt (lane **1**), H358 naïve cells (lane **2**), H1299 naïve cells (lane **3**), H1299 cells expressing 4HC (lane **4**), H1299 cells expressing PTEN4A (lane **5**), and H1299 cells expressing PTENWt (lane **6**). A representative blot from three independent experiments was shown (**A**, **top**). (**B**) The p-PTEN/PTEN ratio in H1299cells expressing 4HC and PTENWt is presented as the intensity level relative to that in H1299 cells expressing PTEN4A (**A**, **bottom**). Data shown represent the means ± SE. The experiment was repeated three times with similar results. *: p<0.05 (**C**) H1299 cells expressing 4HC, PTEN4A, or PTENWt in the absence or presence of TGFβ stimulation were harvested for the analysis of vimentin, ZO-1, and β-actin by western blotting. The relative expression of vimentin to ZO-1 (V/Z ratio) is shown in comparison to that in H1299 cells expressing 4HC treated with vehicle. A representative blot from three independent experiments is shown. Data shown represent the means ± SE. The experiment was repeated three times with similar results. *: p<0.05 N.S. indicates “not significant”. (**D**) A migration assay was performed for H1299 cells expressing 4HC, PTEN4A, or PTENWt in the absence or presence of TGFβ stimulation. Data shown represent the means ± SD. The experiment was repeated three times with similar results. *: p<0.05 (**E**) H1299 cells expressing 4HC, PTEN4A, or PTENWt were cultured for 24hours in the absence or presence of TGFβ stimulation. Snail mRNA was analyzed and normalized to GAPDH mRNA, by using real-time PCR. The relative expression of the snail gene is shown in comparison to that in cells treated with vehicle in absence of Dox. Data shown represent the means ± SEM from three independent experiments. *: p<0.05 (**F**) Cell extracts from H1299 cells expressing 4HC, PTEN4A, or PTENWt in the absence or presence of TGFβ stimulation were harvested for analysis of the levels of total and phosphorylated for smad2, Akt473, Akt308, and FAK at the indicated periods after treatment with vehicle or TGFβ (1hour for smad2, 1hour for Akt473, 1hour for Akt308, and 24hours for FAK, respectively). A representative blot from three independent experiments is shown. (**G**) The ratio of phosphorylated protein to total protein is presented as the intensity level relative to that in H1299 cells expressing 4HC treated with vehicle (S2; smad2, A; Akt, F; FAK, respectively). Data shown represent the means ± SE. The experiment was repeated three times with similar results. *: p<0.05 N.S. indicates “not significant”. The fluorescence intensity of β-catenin in H1299 cells expressing 4HC (**H**), PTEN4A (**J**), or PTENWt (**L**) were evaluated. Nuclear staining was performed by Hoechst33342. Each left image in (**H**), (**J**), and (**L**) shows cells with no TGFβ stimulation. Each right image in (**H**), (**J**), and (**L**) shows cells stimulated with TGFβ. Each upper panel in (**I**), (**K**), and (**M**) plots the fluorescence intensity of β-catenin (red) and nucleus (blue) over a cross section of cells with no TGFβ stimulation. Each lower panel in (**I**), (**K**), and (**M**) plots the fluorescence intensity of β-catenin (red) and nucleus (blue) over a cross section of cells stimulated with TGFβ. These figures are representative of at least three independent experiments. (**N**) Cell proliferation was measured by WST assay for H1299 cells expressing 4HC, PTEN4A, or PTENWt untreated with TGFβ. The experiment was repeated three times with similar results. (**O**) Cell proliferation was also measured for H1299 cells expressing 4HC, PTEN4A, or PTENWt treated with TGFβ. The experiment was repeated three times with similar results. N.S. indicates “not significant”. *: p<0.05.

To validate that modulation of phosphorylation sites in the PTEN C-terminus can negatively regulate TGFβ-induced aberrant activities such as EMT and cell motility in lung cancer cells, another line of lung cancer cells, H1299 cells, was evaluated. The p-PTEN/PTEN ratio was increased in H1299 cells treated with TGFβ for both 24 hours and 48 hours (data not shown). To confirm the ability of PTEN4A to inhibit TGFβ-induced EMT in lung cancer cells, H1299 cells ectopically expressing 4HC, PTEN4A, or PTENWt were established (Figure 7A). The p-PTEN/PTEN ratio in H1299 cells expressing PTEN4Awas significantly lower than that in cells expressing 4HC or PTENWt (Figure 7B). TGFβ treatment induced more than a two-fold increase in the vimentin/ZO-1 ratio in H1299 cells expressing 4HC, and ectopic expression of PTEN4A inhibited this TGFβ-induced increase in the vimentin/ZO- 1 ratio (Figure 7C). Ectopic expression of PTEN4A repressed the ability of H1299 cells to migrate toward a chemoattractant after TGFβ treatment, as compared with cells ectopically expressing 4HC (Figure 7D). TGFβ stimulation induced a significant increase in snail expression in H1299 cells expressing control 4HC, PTENWt, or PTEN4A (Figure 7E). The effect of PTEN4A on TGFβ-induced signaling pathways was also evaluated in H1299 cells. Ectopic expression of PTEN4A did not appear to inhibit TGFβ-induced activation of the smad2 signaling pathway, whereas it significantly inhibited TGFβ-induced smad-independent pathways, including Akt and FAK (Figure 7F and 7G). To determine whether or not TGFβ can modulate β-catenin translocation from the cell membrane into the cytoplasm and the nucleus via phosphorylation of the PTEN C-terminus, localization of β-catenin was evaluated in TGFβ-treated lung cancer cells by immunofluorescence. β-catenin was localized on the cell membrane in H1299 cells ectopically expressing PTEN4A and PTENWt (Figure 7J-7M), whereas it was diffusely observed in the cytoplasm in cells expressing 4HC (Figure 7H and 7I). Although TGFβ stimulation induced translocation of β-catenin into the cytoplasm and the nucleus in H1299 cells expressing PTENWt (Figure 7L and 7M), β-catenin remained localized on the cell membrane after TGFβ stimulation in H1299 cells expressing PTEN4A protein (Figure 7J and 7K). When the effect of mutation of phosphorylation sites in PTEN on cell proliferation was evaluated, the WST-1 assay showed that both GFP-PTENWt and GFP-PTEN4A induced a significant decrease in cell proliferation in H1299 cells treated with TGFβ as compared with GFP (Figure 7N and 7O).

### Mutation of phosphorylation sites in the PTEN C-terminus represses tumor growth in vivo

**Figure 8:**
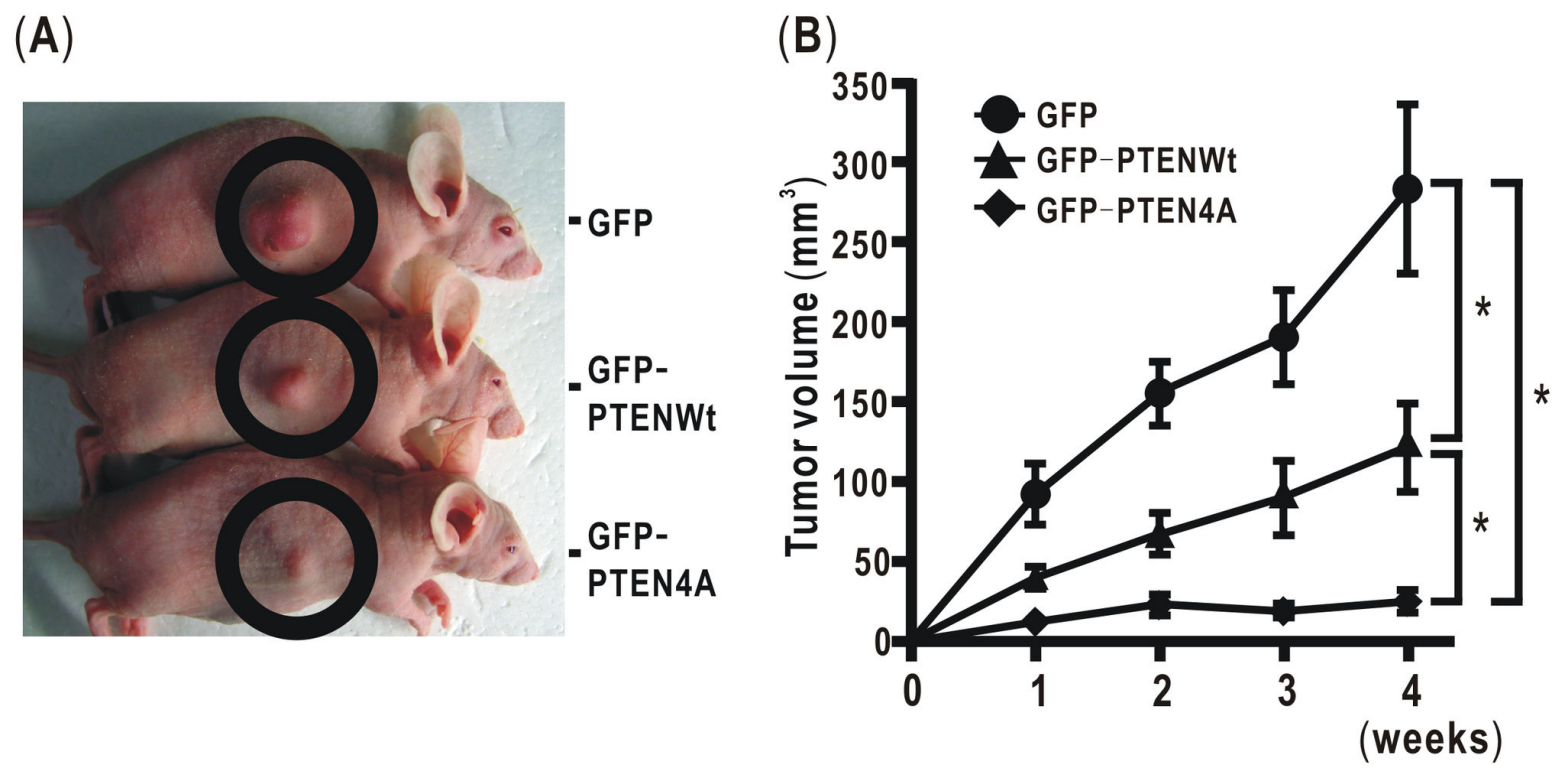
Mutation of phosphorylation sites in the PTEN C-terminus represses tumor growth in H358 cells in vivo. 3x10^6^ H358ON cells expressing Dox-dependent GFP, GFPPTENWt, or GFPPTEN4A, were inoculated subcutaneously into the flank of 6-week-old female nude mice, which were then maintained on water containing Dox at a final concentration of 2mg/ml and autoclaved feed ad libitum. Tumor size was monitored for 4weeks in Dox-treated nude mice. (**A**) A representative example of nude mice with growing tumors at 4weeks after inoculation is shown (H358ON expressing GFP in **top**, and GFPPTENWt in **middle**, and GFPPTEN4A in **bottom**). Tumors were observed within the black circle. (**B**) The tumor growth rate of each cell was evaluated for 4 weeks after inoculation. *: p<0.05 Data shown represent the means ± SEM of three independent experiments. Each experiment used 5 nude mice for GFPPTENWT, 7 nude mice for GFP, and 7 nude mice for GFPPTEN4A.

To evaluate whether mutation of phosphorylation sites in PTEN can modulate tumor growth *in vivo*, H358ON cells expressing Dox-dependent GFP, GFP-PTENWt, or GFP-PTEN4A were inoculated into the flank of nude mice on a BALB/C background. Dox treatment commenced on day 0 with cell inoculation, and then tumor size was monitored for 4weeks. In mice inoculated with H358ON cells expressing GFP, large tumors grew in the flank; by contrast, in mice inoculated with H358ON cells with GFP-PTEN4A, tumors were barely observed even after 4 weeks (Figure 8A). The tumor volume in GFP-PTEN4A-inoculated mice was significantly smaller than that in GFP-inoculated or GFP-PTENWt-inoculated mice (Figure 8B), indicating that four-Ala substitution of phosphorylation sites in the C-terminus of PTEN expressed in tumors inhibited tumor growth *in vivo*, in part due to the combined effects shown in the above *in vitro* experiments.

## Discussion

A previous biochemical analysis demonstrated that phosphorylation of the PTEN C-terminus, leading to a conformational change of its closed phosphatase domain [14], might yield not only a loss of membrane binding but also a down-regulation of PTEN phosphatase activity [13,14]. In addition, recent studies have demonstrated that phosphorylation levels of PTEN are significantly higher in malignant leukemia cells than in normal B cells [35,36]. Those studies also suggest that phosphorylation of the PTEN C-terminus may lead to loss of its function as a phosphatase in malignant leukemia cells [35,36]. Thus, the PTEN C-terminus might be a promising target for regulation of PTEN activity. However, whether TGFβas a tissue-stiffening factor derived from cellular components of the microenvironment, can modulate phosphorylation of the PTEN C-terminus in lung cancer cells remains elusive. In the present study, persistent TGFβ stimulation repressed the level of total PTEN protein [15,16] and slightly but substantially increased the level of PTEN phosphorylation in lung cancer cells, yielding a significant increase in the p-PTEN/PTEN ratio. To confirm the direct effects of TGFβ stimulation on modulation of the p-PTEN/PTEN ratio, we used the inhibitor SB 431542 [32]. Our data suggested that inhibition of TGFβ signaling by SB 431542 blocked the TGFβ-induced increase in the p-PTEN/PTEN ratio. Our data also demonstrated that persistent stimulation with TGFβ induced increasing cell motility and the acquisition of EMT phenotypes via the translocation of β-catenin into the cytoplasm and the nucleus in lung cancer cells, compatible with the results of previous studies, in which fibronectin and E-cadherin are involved in biomarkers of EMT [4,28-30]. Furthermore, TGFβ stimulation induced not only smad2 phosphorylation but also the activation of smad-independent signaling pathways, including phosphorylation of FAK and Akt [6]. Taken together [35,36], the present data suggest that TGFβ may repress total PTEN expression and phosphorylation of PTEN, not only inhibiting PTEN activity but also promoting the activation of β-catenin and signaling pathways.

To elucidate a critical role of phosphorylation of the PTEN C-terminus in acquisition of TGFβ-induced malignant phenotypes, we established H358ON cells – lung cancer cells with a Dox-dependent gene expression system- in which GFP, GFP-PTENWt, or GFP-PTEN4A expression was induced only when Dox was added. A recent study has reported that unphosphorylated PTEN with an open conformation is subject to ubiquitination, which accelerates its degradation and translocation into the nucleus [21]. In the present study, however, the subcellular distribution of unphosphorylated PTEN (PTEN4A) and PTENWt did not differ in H358ON cells. We evaluated the effect of compensatory induction of PTEN4A on TGFβ-induced malignant phenotypes. Our data suggested that *de novo* expressed GFP-PTEN4A protein exhibited an approximately two-fold greater ability to repress TGFβ-induced EMT, as compared with *de novo* expressed GFP-PTENWt protein. Furthermore, *de novo* expressed GFP-PTEN4A protein, but not GFP or GFP-PTENWt protein, repressed TGFβ-induced cell migration in H358ON cells. In contrast, both PTENWt and PTEN4A repressed TGFβ-induced cell migration in H1299 cells, as compared with 4HC. Previous studies have demonstrated that the response to PTENWt transduction might depend on both PTEN gene mutation and PTEN expression levels in glioma cells [15,37,38]. In the present study, H1299 cells showed much lower expression of endogenous PTEN as compared with H358 cells. Thus, the differential effects of PTENWt transduction on TGFβ-induced cell migration might be due to differing endogenous levels of PTEN among lung cancer cells. The expression of the EMT markers varies among cancer cells [3,28,29]. Because no or little expression of fibronectin and E-cadherin is observed in H1299 cells [39], we evaluated TGFβ-induced modulation of vimentin and ZO-1. A significant increase in vimentin/ZO-1 ratio induced by TGFβ was observed in H1299 cells expressing 4HC and PTENWt, whereas the increase in vimentin/ZO-1 ratio was inhibited in H1299 cells expressing PTEN4A. Thus, it should be emphasized that compensatory induction of PTEN4A repressed TGFβ-induced EMT and cell motility in lung cancer cells, regardless of endogenous PTEN expression. 

To elucidate the underlying molecular mechanisms, we evaluated the effect of PTEN4A on TGFβ-induced signaling pathways. Although a recent study has suggested that transcription of EMT target genes might be activated by cytoplasmic β-catenin and lymphoid enhancer factor (LEF)-1 complexes, which TGFβ/smad2 signaling might up-regulate [4], our data indicated that induction of PTEN4A did not modulate TGFβ-induced smad2 phosphorylation. In the present study, the EMT phenotype induced by TGFβ was not completely restored when PTEN4A was induced (Figure 2D), which might be partially due to the TGFβ-induced smad-dependent signaling pathway. Compensatory induction of PTEN4A had greater effects on TGFβ-induced phosphorylation of Akt, as compared with PTENWt; nevertheless, compensatory induction of both PTENWt and PTEN4A significantly repressed TGFβ-induced phosphorylation of Akt to basal levels as compared with the control. Although TGFβ stimulation might be associated with phosphorylation of FAK at Tyr397 [40], our data demonstrated that PTEN4A, but not PTENWt, inhibited TGFβ-induced FAK phosphorylation at Tyr397. PTEN is assumed to be the main negative regulator of the PI3K-Akt pathway [41], and although PTEN is also a negative regulator for FAK activity [42], FAK activation is regulated by many signaling pathways such as Src and integrins [43,44]. Although compensatory induction of PTENWt might be enough to inhibit TGFβ-induced Akt signaling pathways after TGF stimulation, it appeared to be insufficient to inhibit TGFβ-induced FAK phosphorylation, which might depend on TGFβ-induced phosphorylation levels of the PTENWt C-terminus. Thus, our data demonstrated that compensatory induction of PTEN4A comprehensively repressed TGFβ-induced activation of the Akt and FAK signaling pathways, but not the smad-dependent pathway. Compensatory induction of PTENWt also inhibited PI3K signaling, whereas it only partially inhibited TGFβ-induced EMT. This finding is compatible with previous studies showing that both LY294002, a PI3K/Akt inhibitor, and rapamycin, an mTOR specific inhibitor, block aberrant cell motility but do not rescue EMT [32,45]. Thus, our observed repression of TGFβ-induced EMT does not appear to be due to inhibition of Akt/PI3K signaling by PTEN4A. A recent study has demonstrated that FAK activation induces the translocation of stabilized β-catenin from the cytoplasm into the nucleus, resulting in targeted gene expressions [24]. In addition, Deng, et al. showed that repression of whole FAK expression, via FAK siRNA, inhibits TGFβ-induced EMT [46]. Nevertheless, whether PTEN4A can block TGFβ-induced EMT and β-catenin translocation from the cell membrane into the cytoplasm via inhibition of FAK activation remains elusive. Our data suggested that inhibition of FAK phosphorylation at Tyr397 by FAK inhibitor 14 blocked TGFβ-induced cell motility [47], but did not block TGFβ-induced EMT or β-catenin translocation into the cytoplasm. Taken together, our data indicate that compensatory PTEN4A expression might inhibit TGFβ-induced EMT, besides its inhibitory effect on TGFβ-induced activation of smad-independent signaling pathways. Although TGFβ stimulation induces snail [48,49], our data suggested that TGFβ-induced snail gene expression was not altered after compensatory induction of PTEN4A in H358ON cells and H1299 cells. A recent study has demonstrated that transduction of ectopic E-cadherin is sufficient to block EMT and high cell motility induced by ectopic snail expression, indicating that repression of *de novo* snail induction might not be necessary to restore EMT [4]. Our data demonstrated that modulating phosphorylation of the PTEN C-terminus via PTEN4A could block TGFβ-induced β-catenin translocation from the cell membrane into the cytoplasm in lung cancer cells, compatible with those recent studies [4,31]. Although the exact mechanism, by which PTEN4A could block TGFβ-induced β-catenin translocation from the cell membrane into the cytoplasm, remains elusive, a previous study has suggested that phosphorylation of β-catenin might induce its translocation via dissociation from E-cadherin complexes [50]. Inhibiting phosphorylation of the PTEN C-terminus might retain PTEN protein phosphatase activity, resulting in both the blockade of β-catenin phosphorylation and TGFβ-induced EMT. Further investigation is warranted. Taken together, these data suggest that compensatory induction of PTEN4A might repress TGFβ-induced EMT *in vitro* through complete blockade of β-catenin translocation into cytoplasm rather than through modification of TGFβ-induced expression of E-cadherin repressor snail. 

In the present study, expression of PTEN4A led to significantly more repression of cell proliferation under TGFβ stimulation, as compared to control GFP and PTENWt, although there was no difference in the proliferation of cells without TGFβ stimulation. These results might imply that compensatory induction of PTEN4A might control cell proliferation only when an exogenous and excessive stimulus such as TGFβ is given in a pathological microenvironment [35], and that PTEN4A might not modulate cell proliferation in cells in a normal microenvironment. Finally, we evaluated the effect of compensatory induction of PTEN4A on tumor growth *in vivo*. Recent studies have demonstrated that growth factors and cytokines/chemokines derived from cellular components such as CAFs and persistent hypoxia in the tumor microenvironment play a critical role in tumor growth [1,17,51]. Our findings might be explained by a mechanism in which TGFβ-induced complex signal networks are inhibited by *de novo* PTEN4A expression [3,35]. Although we did not evaluate TGFβ-induced modulation of phosphorylation of the PTEN C-terminus in non-malignant epithelial cells, recent studies have also suggested that tissue fibroblasts with decreased PTEN activity might accelerate the development of tumors, indicating the importance of PTEN activity in not only tumor cells themselves but also cellular components of the tumor microenvironment [1,51]. Our data indicated that ectopic induction of PTEN4A into both tumor and other cells including fibroblasts in the tumor microenvironment might directly and indirectly repress the development of tumors. Research into exogenous administration of the PTEN4A gene *in vivo* is warranted.

Overall, our data clearly showed that compensatory induction of PTEN4A had greater effects *in vitro* and *in vivo*, as compared with PTENWt. Although compensatory induction of PTENWt showed a partial inhibitory effect on TGFβ-induced acquisition of malignant phenotypes, PTENWt remained subject to TGFβ-induced phosphorylation of its PTEN C-terminus, resulting in a loss of activity of PTEN, as compared with PTEN4A. A recent study has suggested that translocation of β-catenin into the nucleus might confer resistant to Akt inhibitors in colon cancers [52]. Our data suggested that complete blockade of TGFβ-induced translocation of β-catenin from the cell membrane into the cytoplasm occurred in both H358 and H1299 cells expressing PTEN4A but not PTENWt. PTENWt might undergo TGFβ-induced phosphorylation of the PTEN C-terminus, resulting in destabilization of E-cadherin/β-catenin complexes in the cell membrane. Previous reports have suggested that pleiotropic serine/threonine protein kinases such as casein kinase 2 (CK2) and microtubule-associated Ser/Thr kinases might regulate phosphorylation of serine/threonine sites in the PTEN C-terminus [35,53]. However, other studies show that CK2 inhibitors such as 4,5,6,7-tetrabromobenzotriazole (TBB) and apigenin might induce apoptosis in many cells [54,55], compatible with data suggesting that more than 300 proteins are substrates for CK2 [54,56,57]. Therefore, research into a specific inhibitor of the phosphorylation sites in the PTEN C-terminus is warranted.

In summary, our study is the first to demonstrate that phosphorylation sites in the PTEN C-terminus might be a promising therapeutic target to negatively regulate TGFβ-induced aberrant activities such as EMT, cell motility, and aggressive tumor growth in lung cancer cells. 
